# Generation of Color Images by Utilizing a Single Composite Diffractive Optical Element

**DOI:** 10.3390/mi9100508

**Published:** 2018-10-09

**Authors:** Jiazhou Wang, Liwei Liu, Axiu Cao, Hui Pang, Chuntao Xu, Quanquan Mu, Jian Chen, Lifang Shi, Qiling Deng

**Affiliations:** 1 Institute of Optics and Electronics, Chinese Academy of Sciences, Chengdu 610209, China; wangjiazhou0401@163.com (J.W.); a1252909630@163.com (L.L.); longazure@163.com (A.C.); wuli041@126.com (H.P.); xuchuntao@live.com (C.X.); dengqiling@ioe.ac.cn (Q.D.); 2 School of Optoelectronics, University of Chinese Academy of Sciences, Beijing 100049, China; 3 State Key Laboratory of Applied Optics, Changchun Institute of Optics, Fine Mechanics and Physics, Chinese Academy of Sciences, Changchun 130033, China; muquanquan@ciomp.ac.cn; 4 State Key Laboratory of Transducer Technology, Institute of Electronics, Chinese Academy of Sciences, Beijing 100190, China; chenjian@mail.ie.ac.cn

**Keywords:** diffractive optics, gratings, microfabrication, computer holography

## Abstract

This paper presents an approach that is capable of producing a color image using a single composite diffractive optical element (CDOE). In this approach, the imaging function of a DOE and the spectral deflection characteristics of a grating were combined together to obtain a color image at a certain position. The DOE was designed specially to image the red, green, and blue lights at the same distance along an optical axis, and the grating was designed to overlay the images to an off-axis position. We report the details of the design process of the DOE and the grating, and the relationship between the various parameters of the CDOE. Following the design and numerical simulations, a CDOE was fabricated, and imaging experiments were carried out. Both the numerical simulations and the experimental verifications demonstrated a successful operation of this new approach. As a platform based on coaxial illumination and off-axis imaging, this system is featured with simple structures and no cross-talk of the light fields, which has huge potentials in applications such as holographic imaging.

## 1. Introduction

A diffractive optical element (DOE) is a phase modulation element that has the potential to significantly promote the miniaturization, integration, and arrangement of conventional optical systems [1,2,3,4,5,6]. More specifically, DOEs play a key role in the field of holographic imaging [7,8]. However, the working wavelengths of traditional DOEs are monochromatic; thus, multiple DOEs are always needed in the holographic imaging of color pictures, leading to complex and bulky systems.

In order to address this issue, previous studies were conducted to generate color images using one single DOE, which relied on coaxial imaging and coaxial illumination [9,10,11,12,13,14,15,16,17] or coaxial imaging and off-axis illumination [18,19,20]. In coaxial imaging and coaxial illumination, the phase modulations of incident lights with different wavelengths are obtained by properly designing DOEs. More specifically, Bengtsson proposed an algorithm that can produce a specific image of two wavelengths at a certain distance [9,10,11]. Ogura et al. optimized the weighting factors of this algorithm to realize multi-wavelength imaging [12,13]. Jesacher et al. proposed the concept of equivalent phases to optimize the designs of DOEs, which mainly relied on the modification of the Gerchberg–Saxton (GS) algorithm [14,15]. However, these approaches suffered from the limited numbers of imaging points, close distances of output fields, and complex fabrications of DOEs. 

Meanwhile, DOEs were also designed using imaging features of the Fresnel DOE, where the imaging position varies for multi-wavelength incident lights to obtain color holograms [16,17]. However, the proposed optical field was prone to cross-talk among the individual color components. In order to address this issue, coaxial imaging and off-axis illumination were proposed to generate color images using one single DOE, where the RGB (red, green, and blue) components of color images are encoded at different positions within the same plane, and thus the superposition of the light field can be realized by changing the irradiation angles of the incident lights [18,19,20].

However, since the incident angles of each wavelength’s components need to meet specific design requirements, a complicated mechanical structure and a bulky system were required in this approach. In addition, slight deviations of the angles of the incident lights can affect the quality of the color images, and thus the stability of the proposed systems was under question. 

In order to deal with the aforementioned problems, we report in this paper the design of a composite diffractive optical element (CDOE) for generating color images. This design offers significant improvements in comparison to the traditional approach based on coaxial incidences and off-axis imaging to generate color images, whereby (1) only a RGB mixed light was used as the incident light on the CDOE, significantly reducing the complexity of the optical system; and (2) the principle of spatial superposition was adopted, ensuring that the obtained target field was well recognizable. The structures of this paper are as follows: 1. Introduction, 2. Principle, 3. Simulation, 4. Experiments, and 5. Summary.

## 2. Principle

### 2.1. Schematics

**Figure 1:**
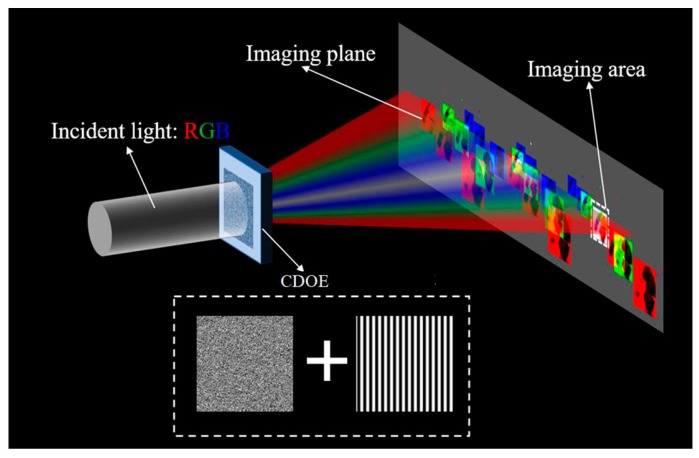
The schematic of an imaging method by using composite diffractive optical elements (CDOE) comprising a diffractive optical elements (DOE) and a grating.

The principle of this method is illustrated in Figure 1, where the CDOE is irradiated by a composite RGB light beam along an optical axis, generating a color image at off-axis positions. The CDOE proposed in this study contained the modulation phase to generate the target field composing RGB components of the color images, and the phase information of the spectral grating, which was used to modulate the three light fields to a specific position for the superposition. The design process and the parameter analysis will be discussed carefully in the following sections.

### 2.2. The Process of Designing and Parameter Analysis 

The amplitude distribution of the target color light was denoted by *A*(*x*, *y*). Since all of the colors can be synthesized by RGB components, the target color field could be represented by RGB components, in which the individual amplitude distributions of the three color components were denoted by *A_R_*(*x*, *y*),*A_G_*(*x*, *y*), and *A_B_*(*x*, *y*), respectively.

**Figure 2:**
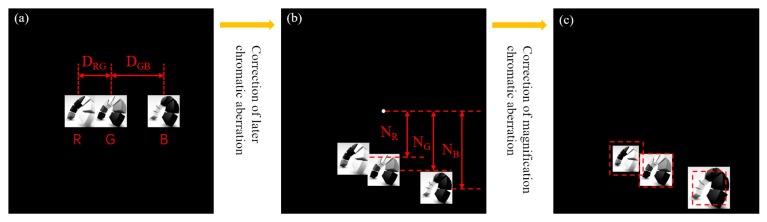
(**a**) The position shifts of red, green, and blue (RGB) components according to the parameters D_RG_ and D_GB_; (**b**) the correction for lateral chromatic aberration; (**c**) the correction for magnification chromatic aberration.

In the design process, the chromatic aberrations of the RGB lights were addressed. The revised amplitude distributions *A’_R_*(*x*, *y*),*A_G_*(*x*, *y*), and *A’_B_*(*x*, *y*) were obtained by making compensations to the R and the B components, while the G component remained unchanged. The three distributions were arranged from left to right in the design plane, and the distances between each of the two components were represented as *D_RG_* and *D_GB_,* respectively, as shown in Figure 2a.

By combining *A’_R_*(*x*, *y*),*A_G_*(*x*, *y*), and *A’_B_*(*x*, *y*) together, the target amplitude distribution *E_m0_*(*x*, *y*) was obtained first. A DOE was then designed for the green light in order to obtain a phase distribution of *ϕ_G_*(*x*, *y*). When the RGB lights passed through the DOE, the light fields of *E_G_*(*x*, *y*), *E_R_*(*x*, *y*), and *E_B_*(*x*, *y*) were obtained, respectively. However, the three light fields were not imaged in the correct position to form a color image. So, a grating phase *ϕ_T_*(*x*, *y*) was designed and superimposed on *ϕ_G_*(*x*, *y*) to alter the positions of the three light fields, whose periods determined the values of the *D_RG_* and *D_GB_*. The accurate modulations of the RGB lights were realized by carefully choosing the interactions of *ϕ_G_* and *ϕ_T_*.

A superposition of *E_R_*(*x*, *y*), *E_G_*(*x*, *y*), and *E_B_*(*x*, *y*) created a color image at the first-order position of the green light. In the design process, the parameters determining *D_RG_* and *D_GB_*, the corrections of chromatic aberrations, and the interactions between *ϕ_G_* and *ϕ_T_* were analyzed in detail in order to obtain the final amplitude distribution *A*(*x*, *y*). In addition, the impacts of the zero-level positions and its optimization measures were considered carefully.

#### 2.2.1. Determination of the Parameters *D_RG_* and *D_GB_*

In the design process, the target field of the DOE was obtained by coding the three sets of amplitude distributions of the RGB components. Then, the issue was how to determine the parameters of *D_RG_* and *D_GB_*, which express the intervals between amplitudes. The intervals were cooperated with the wavelength-dependent spectral deflection abilities of the grating to realize the superposition of the light fields. Thus, the values of *D_RG_* and *D_GB_* were closely related to the grating periods. At the same time, three amplitude distributions were generated by the DOE. Therefore, the values were also closely related to the structural parameters of the DOE, with the detailed analysis discussed below. 

Suppose that the period of grating was *d*, and the spectral deflection ability of grating was expressed by the grating equation:

(1) $$\theta =\arcsin\left(\frac{m\lambda }{d}\right)\;$$

where *λ* is the wavelength of the incident light, and *θ* is the angle between the m-order of the light and optical axis in the imaging plane. The distance between the imaging plane and the DOE was *z*. The sampling interval *δ* of the imaging plane was closely related to the period *D* of the DOE, which was expressed by the following equation:

*δ* = *λz*/*D* (2)

Both the sampling interval *δ* and the angle *θ* depend on light wavelength. Thus, the position of m-order light generated by the grating depends also on light wavelength. For convenience, a pixel number *M* was used to express the position of m-order light (relative to that of the zero-order light), which was expressed by the following equation:

(3) $$M=\frac{\tan\left(\theta \right)z}{\delta }\;$$

The position of the m-order green light was taken as the image area. Since the angle *θ* between the image area and the axis was very small, Equation (3) was reduced to Equation (4) in case of paraxial:

(4) $$M=\frac{mD}{d}\;$$

The pixel numbers for each of the three RGB wavelength components were expressed by Equation (5):

(5) $$M_{R}=\frac{mD\lambda_{G}}{d\lambda_{R}};M_{G}=\frac{mD}{d};M_{G}=\frac{mD\lambda_{G}}{d\lambda_{B}}\;$$

The green light wavelength was regarded as the nominal working wavelength of the DOE, and the green component of the color image was coded in the middle of the target field. Thus, the intervals *D_RG_* and *D_GB_* were expressed as the difference of pixel numbers (*M_G_–M_R_*) and (*M_B_–M_G_*), respectively (see Figure 2a):

(6) $$\begin{array}{l} D_{RG}=\frac{mD}{d}-\frac{mD\lambda_{G}}{d\lambda_{R}}\\ D_{GB}=\frac{mD\lambda_{G}}{d\lambda_{B}}-\frac{mD}{d} \end{array}$$

#### 2.2.2. Correction for the Chromatic Aberration

In order to avoid the influence of zero-order on the color image, the position of the color image was shifted vertically, and the shifting distance was represented as *L*, ensuring that the zero-level does not appear on the image. As can be seen from Section 2.2.1, the sampling interval *δ* of the imaging plane was related to the incident wavelength, and thus, there was a lateral chromatic aberration, which needed to be corrected (see Figure 2b). The amount of shift in pixel numbers for each wavelength component was calculated with the help of Equation (2), as expressed by Equation (7):

(7) $$N_{R}=\frac{LD}{\lambda_{R}z};N_{G}=\frac{LD}{\lambda_{G}z};N_{B}=\frac{LD}{\lambda_{B}z}\;$$

There was also a magnification of the chromatic aberration due to the difference of the sampling interval. The imaging sizes of RGB lights were different from each other. In order to ensure the perfect superposition of each component, it was necessary to adjust the pixel number *X·Y* of the RGB components to ensure that the sizes of each image were the same. The scaling relation was related to δ, and in the case that the z and *D* were constants, they can be expressed by Equation (8):

(8) $$\begin{array}{l} X_{R}:X_{G}=Y_{R}:Y_{G}=\lambda_{G}:\lambda_{R};\\ X_{B}:X_{G}=Y_{B}:Y_{G}=\lambda_{G}:\lambda_{B}; \end{array}$$

where *X_R_·Y_R_*, *X_G_·Y_G_*, and *X_B_·Y_B_* were the corrected pixel numbers of RGB components, respectively. According to the aforementioned analysis, the color image was firstly decomposed into RGB components. The RGB components were then encoded and scaled in the same plane according to the calculations. Finally, the desired target field of the DOE was obtained as shown in Figure 2c.

#### 2.2.3. Interaction Mechanism between Modulation Phase *ϕ_G_* and Grating Phase *ϕ_T_*

**Figure 3:**
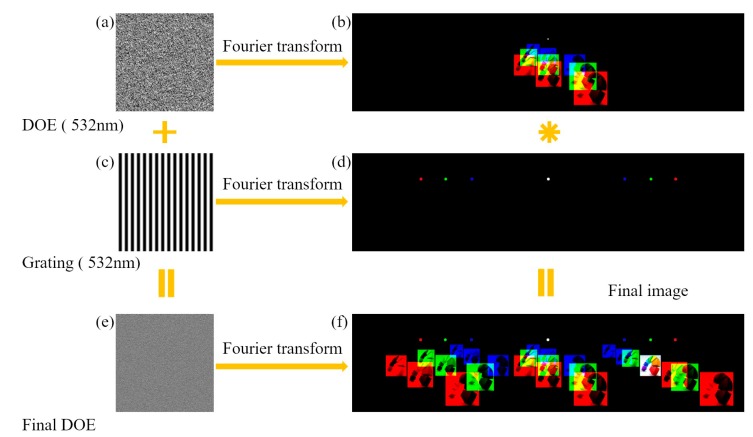
The schematic diagram of the interaction mechanism between modulation phase *ϕ_G_* and grating phase *ϕ_T_*. The phase distribution of (**a**) DOE, (**c**) grating, and (**e**) the final composite DOE. Their corresponding imaging effects are shown in images (**b**), (**d**), and (**f**), respectively.

In this work, the distribution of the modulation phase *ϕ_G_* was calculated by the GS algorithm, as shown in Figure 3a, and the corresponding grating phase *ϕ_T_* (see Figure 3c) was superimposed on the *ϕ_G_* to generate the final phase distribution of the DOE (see Figure 3e). When the RGB lights were regarded as incident lights, the light field of *ϕ_G_* and *ϕ_T_* in the imaging plane were expressed as *E*_1_ and *E*_2_, respectively, by Equation (9):

(9) $$\begin{array}{l} E_{1}=\left(\begin{array}{l} E_{R}^{1}\\ E_{G}^{1}\\ E_{B}^{1} \end{array}\right)=\left(\begin{array}{l} FFT\left(A\exp\left(i\frac{\lambda_{G}}{\lambda_{R}}\varphi_{G}\right)\right)\\ FFT\left(A\exp\left(i\varphi_{G}\right)\right)\\ FFT\left(A\exp\left(i\frac{\lambda_{G}}{\lambda_{B}}\varphi_{G}\right)\right) \end{array}\right)\\ E_{2}=\left(\begin{array}{l} E_{R}^{2}\\ E_{G}^{2}\\ E_{B}^{2} \end{array}\right)=\left(\begin{array}{l} FFT\left(A\exp\left(i\frac{\lambda_{G}}{\lambda_{R}}\varphi_{T}\right)\right)\\ FFT\left(A\exp\left(i\varphi_{T}\right)\right)\\ FFT\left(A\exp\left(i\frac{\lambda_{G}}{\lambda_{B}}\varphi_{T}\right)\right) \end{array}\right) \end{array}$$

where *A* is the amplitude distribution of the incident light, which is a constant for the uniform parallel light. The resulting light field distributions were shown in Figure 3b,d. By superimposing the two phases to obtain the final phase distribution, the corresponding light field *E* was expressed by Equation (10):

(10) $$\begin{array}{l} E=\left(\begin{array}{l} FFT\left(A\exp\left(i\frac{\lambda_{G}}{\lambda_{R}}\left(\varphi_{G}+\varphi_{T}\right)\right)\right)\\ FFT\left(A\exp\left(i\left(\varphi_{G}+\varphi_{T}\right)\right)\right)\\ FFT\left(A\exp\left(i\frac{\lambda_{G}}{\lambda_{B}}\left(\varphi_{G}+\varphi_{T}\right)\right)\right) \end{array}\right)=\left(\begin{array}{l} FFT\left(A\exp\left(i\frac{\lambda_{G}}{\lambda_{R}}\varphi_{G}\right)\cdot \exp\left(i\frac{\lambda_{G}}{\lambda_{R}}\varphi_{T}\right)\right)\\ FFT\left(A\exp\left(i\varphi_{G}\right)\cdot \exp\left(i\varphi_{T}\right)\right)\\ FFT\left(A\exp\left(i\frac{\lambda_{G}}{\lambda_{B}}\varphi_{G}\cdot \exp\left(i\frac{\lambda_{G}}{\lambda_{B}}\varphi_{T}\right)\right)\right) \end{array}\right)\;\\ =A\cdot \left(\begin{array}{l} FFT\left(\exp\left(i\frac{\lambda_{G}}{\lambda_{R}}\varphi_{G}\right)\right)*FFT\left(\exp\left(i\frac{\lambda_{G}}{\lambda_{R}}\varphi_{T}\right)\right)\\ FFT\left(\exp\left(i\varphi_{G}\right)\right)*FFT\left(\exp\left(i\varphi_{T}\right)\right)\\ FFT\left(\exp\left(i\frac{\lambda_{G}}{\lambda_{B}}\varphi_{G}\right)\right)*FFT\left(\exp\left(i\frac{\lambda_{G}}{\lambda_{B}}\varphi_{T}\right)\right) \end{array}\right)=A\cdot \left(\begin{array}{l} E_{R}^{1}*E_{R}^{2}\\ E_{G}^{1}*E_{G}^{2}\\ E_{B}^{1}*E_{B}^{2} \end{array}\right)\; \end{array}$$

where * means convolution. It was noticed from Equation (10) that the final light field *E* can be obtained by the convolution of *E*_1_ and *E*_2_. The light field *E*_1_ was repeated in each order of the light field *E*_2_ generated by the grating phase. It was observed that the order position of different incident lights was different from Figure 3d, and the superposition of RGB components was achieved by using this property to complete the generation of color images (see Figure 3f).

## 3. Simulation

**Figure 4:**
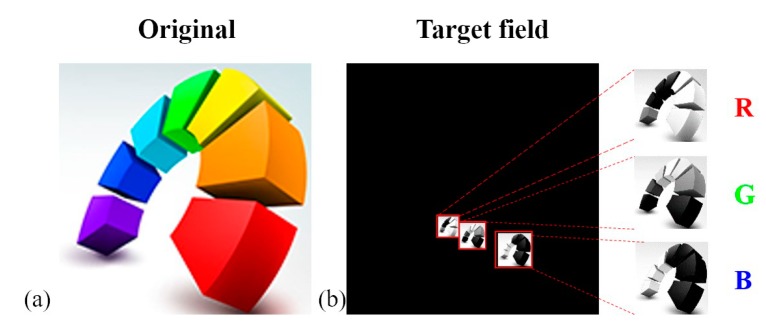
(**a**) The target color field; (**b**) the simulated imaging light field of RGB components.

To validate the design methodologies described above, numerical simulations were conducted to obtain the target images (see Figure 4a). The simulation was carried out by using Matlab-based programs that we wrote. The design of the CDOE and the imaging effect can all be simulated by the programs. The wavelengths *λ_R_*, *λ_G_*, and *λ_B_* that were used in simulation were 650 nm, 532 nm, and 406 nm, respectively. The pixel size of the color target image was 512 × 512, which was decomposed into RGB components, and arranged based on the aforementioned analysis. The imaging position was selected at the first-order position of the green light imaging. Then, the values of the D_RG_ and D_GB_ intervals were quantified as 492 pixels and 842 pixels, respectively, based on Equation (6). To avoid the effect of zero order, a longitudinal offset was conducted with a vertical offset of 200 pixels relative to the green component. The periods, duty ratios of the grating, and phase depth were quantified as 3 μm, 1:1, and π for the green light, respectively. The characteristic feature size of the DOE was 1.5 μm. Based on the above data, a synthetic target field with 5335 × 5335 pixels was obtained. In order to use a CCD to receive the image, a Fourier lens with the focal length of 37.5 cm was added into the system. The simulated imaging light field is shown in Figure 4b.

**Figure 5:**
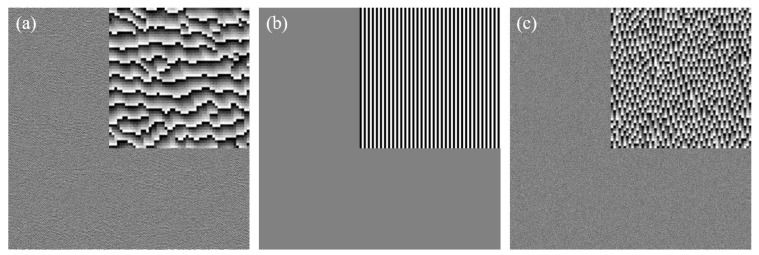
The simulation of the phase distribution: (**a**) the phase distribution of the DOE; (**b**) the phase distribution of the grating; and (**c**) the final composite phase distribution of CDOE.

Using the aforementioned parameters, where 532 nm was used as the working (green) wavelength, the calculated target field was translated to the phase distribution of the single-wavelength DOE. As shown in Figure 5a, the grating phase distribution critical feature size of 1.5 μm, and periods of grating of 3 μm as shown in Figure 5b were superimposed on the DOE phase to obtain the final composite phase distribution of the CDOE as shown in Figure 5c with a level height of 1.156 μm.

**Figure 6:**
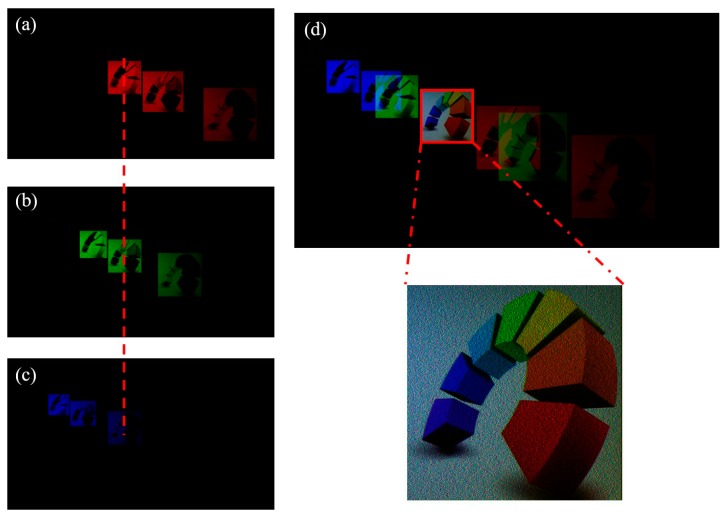
Simulation results: (**a**), (**b**), and (**c**) are the imaging light field of red, green, and blue incident lights, respectively; (**d**) the composite color imaging light field.

**Figure 7:**
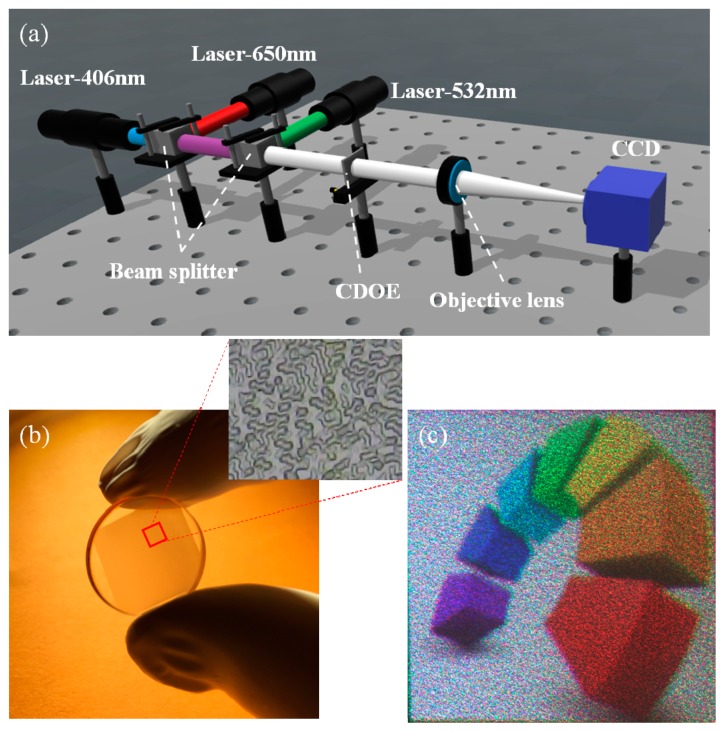
(**a**) Experimental setup of the coaxial illumination system to generate color images and experiment results by using the fabricated CDOE; (**b**) the photograph of the fabricated composite diffractive optical element; (**c**) the generated color image.

The simulated imaging results are shown in Figure 6, with (a), (b), and (c) representing the imaging fields generated by red, green, and blue lights passing through the DOE, respectively. Each light field has an amplitude distribution of RGB components, and the central position of three images was used for imaging combination, producing the color images shown in Figure 7d. The generated color images were consistent with the target image, indicating a high degree of color reproduction, and validating the feasibility of this approach to produce full color images.

## 4. Experiments

To further validate the feasibility of the proposed approach, corresponding experiments were carried out. Firstly, the CDOE was fabricated by using the photolithography. Silica was chosen as the substrate, and AZ9260 was chosen as the photoresist. The photoresist was spin-coated on the substrate at a rotation speed of 5000 rad/min. The process parameters of coating time, prebake temperature, prebake time, and the resist thickness were 30 s, 100 °C, 5 min, and 3 μm, respectively. A photolithography system was employed to perform the exposure. The illumination light source was an Hg lamp with a central wavelength of 365 nm. The total exposure time was 20 s. The process parameters of development, after-bake temperature, and after-bake time were 3 min, 120 °C, and 30 min, respectively. Reactive ion etching (RIE) was carried out to transfer the structure into the substrate. The etching gases were SF_6_ and CHF_3_, and the etching time was 80 min. The pictures of the obtained elements are shown in Figure 7b. The light beam paths were constructed as shown in Figure 7a, employing three light sources operated at wavelengths of 460 nm, 532 nm, and 650 nm, respectively. The three parallel beams produced by the laser modules were combined through two beam splitters/combiners (in the form of dispersion prisms) to obtain a mixed light of RGB. The mixed light was irradiated onto the CDOE and imaged by a lens onto a CCD camera. An example image captured by the CCD is shown in Figure 7c, which was consistent with the simulation results, producing nice imaging without optical cross-talk. Furthermore, the incident light is an RGB mixed light functioning as an incident light on the DOE, confirming the coaxiality of the proposed method.

As a phase modulation element, the efficiency of the CDOE is related to the number of levels in the phase construction. The more levels there are, the higher the efficiency. In this method, since the phase level of the CDOE is two, the efficiency of the CDOE is not as high as when using eight or 16 levels. In fact, by using a CDOE with eight or 16 phase levels, the symmetric images can be eliminated, and the efficiency can be increased greatly. In the current research, we focused on the proposal and verification of this new method.

## 5. Summary

This paper presents a novel method to generate color images based on coaxial illumination and off-axis imaging by utilizing a single composite diffractive optical element (CDOE). The principle of this method was analyzed in detail, and the corresponding simulations and experimental implementations were conducted successfully. The simulation and experimental results validated the proposed approach, which featured a simplified system without a cross-talk of light fields. This provides a new perspective for generating color images based on a single CDOE.
